# Neonatal 6-OHDA Lesion Model in Mouse Induces Cognitive Dysfunctions of Attention-Deficit/Hyperactivity Disorder (ADHD) During Young Age

**DOI:** 10.3389/fnbeh.2020.00027

**Published:** 2020-02-26

**Authors:** Otmane Bouchatta, Houria Manouze, Saadia Ba-M’Hamed, Marc Landry, Mohamed Bennis

**Affiliations:** ^1^Laboratory of Pharmacology, Neurobiology and Behavior, Faculty of Sciences, Cadi Ayyad University, Marrakesh, Morocco; ^2^University of Bordeaux, Bordeaux, France; ^3^CNRS UMR 5297, Centre Paul Broca-Nouvelle Aquitaine, Interdisciplinary Institute of Neuroscience, Bordeaux, France

**Keywords:** attention-deficit/hyperactivity disorder, 6-hydroxydopamine, executive functions, latent inhibition, attention, inhibitory control

## Abstract

Attention-deficit/hyperactivity disorder (ADHD) is a syndrome characterized by impaired attention, impulsivity and hyperactivity in children. These symptoms are often maintained in adults. During adolescence, prefrontal cortex develops connectivity with other brain regions to engage executive functions such as, latent inhibition, attention and inhibitory control. In our previous work, we demonstrated the validity of the neonatal 6-Hydroxydopamine (6-OHDA) mouse model, a classical neurodevelopmental model mimicking major symptoms of the human ADHD pathology. In order to evaluate pathological forms of executive functions and impulsive behavior in 6-OHDA mice during young age, we first tested latent inhibition (LI) after weaning, and then we evaluated the impulsive behavior using a cliff avoidance reaction test. Our results demonstrated that 6-OHDA mice showed disruption in latent inhibition, suggesting a deficit in selective attention, and displayed repetitive peering-down behavior, indicating a maladaptive impulsive behavior. Subsequently, to assess impulsivity and attention in young mice, we performed a modified 5-choice serial reaction time task test (5-CSRTT), optimizing the degree of food restriction for young animals and shortening the training duration. This test allowed us to demonstrate a deficit in inhibitory control and a loss of accuracy of 6-OHDA mice in the 5-CSRTT. In conclusion, we demonstrated that the 6-OHDA mouse model reproduces human symptoms of ADHD in childhood and early adulthood periods, as seen in human. Taken together, the 6-OHDA mouse model will be useful alongside other animal models to understand the neurobiological mechanisms underlying complex, heterogeneous neurological disorders.

## Introduction

Attention-deficit/hyperactivity disorder (ADHD) is a developmental disorder identified particularly by hyperactivity, impulsivity and inattention (American Psychiatric Association, 2013) in children. It is well known that ADHD patients exhibit impairments across a range of cognitive abilities, such as learning performance (i.e., latent inhibition), executive functions, novelty-seeking and exploratory activity, and short-term memory (Lubow and Josman, 1993; Faraone and Biederman, 2005; Lubow et al., 2005; Arnsten, 2009; Lubow et al., 2014; Fried et al., 2016). In addition, teens with ADHD exhibit often emotional immaturity and tend to feel more comfortable interact with younger children (Stanford and Tannock, 2012). Their affective status is poorly controlled, they often display exaggerate negative or positive reactions that are unrelated to the situation, and become easily frustrated, irritable and angry (Barkley et al., 2010). ADHD is present in children and continues into adolescence and adulthood in up to half of diagnosed cases (Barkley and Murphy, 1998).

Various animal models have been developed for modeling the neurodevelopmental alterations that occur in ADHD. The most studied ADHD animal models are: spontaneously strained rat (SHR), coloboma mutant mouse, dopamine transporter knockout/down mouse (DAT-KO), and neonatal rat damaged by 6-hydroxydopamine. (i) The use of SHRs as a model of ADHD in the 1990s is linked to their hyperactivity (Wultz et al., 1990; Sagvolden et al., 1992). However, the hyperactivity in the animal is not systematically considered as a model of ADHD (Stanford and Tannock, 2012). Subsequent studies using behavioral tests showed inattention and impulsivity in this SHR model (Evenden and Meyerson, 1999; De Bruin et al., 2003; Jentsch, 2005; Bizot et al., 2007; Fox et al., 2008). (ii) Coloboma mice which have a mutation in the Snap25 sequence are hyperactive (Hess et al., 1996), exhibit also an impairment of latent inhibition, indicating inattention (Lubow and Josman, 1993; Bruno et al., 2007). In addition, this animal model displays also impulsive behavior as demonstrated by delayed reward paradigms which require subjects to choose between an immediately available small reward or a delayed greater reward (Bruno et al., 2007). (iii) In DAT-KO mice, and in addition to the hyperactivity showed by Giros et al. (1996), impulsivity and a decrease in learning performance and memory are described (Gainetdinov et al., 1999; Li et al., 2010). (iv) The neonatal 6-OHDA-lesioned rat model of ADHD has been developed since 1976 by Shaywitz et al. (1976) by selective chemical lesion of dopaminergic neurons in 5-day-old rats. At 2–3 weeks following the lesion, these rats exhibit hyperactivity comparable to that observed in childhood ADHD (Erinoff et al., 1979; Miller et al., 1981; Archer et al., 1988), but there are not impulsive (Arime et al., 2011). Furthermore, hyperactivity in this model has sometimes been associated with inattention (Oke and Adams, 1978; Archer et al., 1988). However, a comprehensive assessment of ADHD-like symptoms is still missing, and data in mouse remain largely unavailable. In our previous work (Bouchatta et al., 2018), we demonstrated the validity of the neonatal 6-OHDA-lesioned mouse model to mimic human ADHD syndrome. At a juvenile stage, they are hyperactive in a novel environment, and exhibit inattention and impulsive-like behavior in adulthood. In addition, we have also shown that this model presents also comorbid symptoms such as learning and memory deficits, antisocial and aggressive behaviors, and a high level of anxiety. However, the executive functions such as latent inhibition, attention and impulsivity have not been systematically investigated during young age in this 6-OHDA model.

Several operant tasks have been developed to assess and highlight the underlying mechanisms of the deficits exhibited by children with ADHD at a preclinical level (Carli et al., 1983; Robbins, 2002). The latent inhibition test is based on the fact that the pre-exposure of a normal animal to a stimulus without reinforcement, makes it indifferent, and delays subsequent conditioning to the same stimulus. This can be explained by an attentional filtration which decreases the attention to an usual stimulus (Matsuo et al., 2009). Impulsivity is characterized by uncontrolled behaviors that are premature, inappropriate and/or irrepressible (Eagle and Baunez, 2010). In animals, the cliff avoidance reaction (CAR) test refers to their innate avoidance reaction to a potential fall from a height. Impaired RCA indicates inadequate impulsive behaviors among adult rodents (Matsuoka et al., 2005; Kumakura et al., 2010; Kuroda et al., 2011) which indicates a deficient behavioral inhibition. The 5-choice serial reaction time task (5-CSRTT) has been used widely to evaluate both attention and impulsivity in adult rodents (Robbins, 2002). It was adapted from Leonard’s five-choice serial reaction task originally designed to assess attentional processes in humans (Wilkinson, 1963). However, the 5-CSRTT cannot be simply extrapolated to adolescent animals for many reasons. First, the tasks usually take months to complete. However, adolescence covers only a few weeks in rodents (Barr et al., 2008), restraining the applicability of this operant task only to adult subjects. Second, the normal food restriction procedure in 5-CSRTT, which is applied to motivate animals to perform the task, could disrupt the normal growth of mice during adolescence and can affect impulsive behavior (Robbins, 2002). For these reasons, the 5-CSRTT needs to be adapted to reliably test impulsivity and inattention in young mice.

The aim of the present study is to evaluate executive functions in 6-OHDA mice between juvenile and young adult periods. We demonstrated a disruption in latent inhibition and impulsive CAR behavior in 6-OHDA juvenile mice. Moreover, we adapted the 5-CSRTT protocol to assess attention and impulsivity during the adolescence-like period in mice. We manipulated the inter-trial interval (ITI) and the stimulus duration (SD) to produce impulsive responding and engage the attention, respectively, upon a stable performance. Young 6-OHDA adult mice showed a significant decrease in accuracy, when attention was tested. Moreover, they also showed more premature responses than sham mice at ITI challenges, indicating a deficit in inhibitory control. In conclusion, our data suggest that young 6-OHDA mice exhibit a comprehensive set of behavioral deficits consistent with ADHD.

## Materials and Methods

### Animals

We used 40 Swiss male mice, bred in the central animal facility of Cadi Ayyad University, Marrakech, Morocco, with water and food *ad libitum*. Pups were housed with their mothers in litters and kept under constant temperature conditions (22°C ± 2), under a 12h light/12 h dark cycle (with light on at 7 am). The study received approval of the Council Committee of the research laboratories of the Faculty of Sciences, Cadi Ayyad University. All procedures were conducted in conformity with the approved institutional protocols and within the provisions for animal care and use prescribed in the scientific procedures on living animals, European Council Directive (EU2010/63). All efforts were made to minimize any animal suffering.

### Neonatal 6-OHDA Lesion at P5

Intracerebroventricular injection of 6-OHDA was performed at P5 in an adapted platform fixed to a stereotaxic instrument (David Kopf instrument, Tujunga, CA, United States) according to Bouchatta et al. (2018) protocol. Briefly, male pups were injected by desipramine hydrochloride (20 mg/kg, s.c.; Sigma-Aldrich, France) as norepinephrine uptake blocker. 30 min later, pups were anesthetized with hypothermia induced by placing pups on ice for 1 min, and then following precise parameters (0.6 mm lateral to the medial sagittal suture, 2 mm rostral to the lambda and 1.3 mm in depth from the skull), they received into one of the lateral ventricles 25 μg of 6-OHDA hydrobromide (Sigma-Aldrich) dissolved in 3 μl of ascorbic acid 0.1%, at 1.5 μl/min, whereas control mice received vehicle. Injections were performed manually using a 30G needle (Carpule, Bayer; Osaka, Japan) connected to a 25 μl Hamilton syringe. After the injection, the pups were warmed up at 37°C, and returned to their mothers until weaning.

### Behavioral Test

**FIGURE 1:**
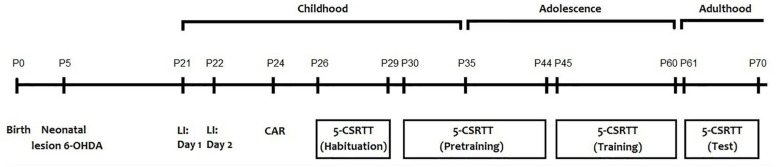
Schematic representation of the experimental design.

All behavioral tests were performed for all animals (sham = 20; 6-OHDA = 20) between 8:00 and 12:00 a.m. to prevent any circadian related fluctuation with the performance of the animals. The behavioral tests were performed as follow: latent inhibition test [postnatal day (PND)21 and 22], cliff avoidance reaction test (PND 24) and the 5-CSRTT (from PND 26 to 70) (Figure 1). Before each test and in order to remove any trace of odor, the apparatus was cleaned with a 75% ethanol solution.

### Latent Inhibition (LI) Test

The protocol was set as previously described (Matsuo et al., 2009). On the first day, each mouse was placed in a training apparatus. Experimental (sham and 6-OHDA) mice were separated into two groups: pre-exposed (P) group and non-pre-exposed (NP) group. The P group (*n* = 10) received 40 white noise tones (55 dB, 5 s duration, 25 s inter-stimulus interval), while the NP group (*n* = 10) received no stimulus during an equivalent period. After, tone-shock associations consisting of a 5 s tone co-terminating with a 2 s foot shock at 0.25 mA were delivered to both groups with a 25 s inter-stimulus interval. All mice were exposed to 3 tone-shock pairings. The mice were submitted to the tone (CS), and during the last second of the tone they received a footshock (US). At 5 min after the CS–US pairing, the CS–US pairing was carried out again. Mice were returned to the home cage 25 s later. On day 2, the mice were placed back in the conditioning chamber for 5 min and the freezing due to the contextual recall was recorded. On the same day, the mice were put in another box (35 × 35 × 40 cm) made of white opaque Plexiglas and after 180 s, a 180 s tone was delivered to measure cued freezing.

### Cliff Avoidance Reaction (CAR) Test

CAR was evaluated using a round wooden platform (diameter 20 cm; thickness 2 cm), fixed on an iron rod 50 cm high (Yamashita et al., 2013). The test was initiated by gently placing the animal on the platform. The CAR was considered altered when the animal fell from the platform and the latency of the fall was recorded. The incidence of altered CAR was calculated as a percentage index for each group:% (CAR) {the number of intact CAR mice (which did not fall from the platforms)/total number of mice tested} × 100. After each fall, the mice were immediately returned to the platform, and the test was continued until 60 min had passed. In mice, which did not fall from the platforms, they were also tested for 60 min.

### 5-Choice Serial Reaction Time Task (5-CSRTT)

#### Apparatus

Mice were trained in computer-controlled operant chambers (24 × 20 × 15 cm) placed inside ventilated sound-attenuating compartment (Med Associates Inc., St. Albans, VT, United States) as described previously (Bouchatta et al., 2018).

#### Initial Handling and Feeding Protocol

Mice underwent 1-min of handling on PNDs 26, 27, 28, and 29 until they are completely habituated to being picked up (Figure 1). Twenty-four hours before the first training session on PND 30, available food was restricted to 1.0 g. During the 5-CSRTT training period, mice were given a diet as follows: providing 2.0 g food (3 weeks old), 2.5 g food (4 weeks old), 2.8 g food (5–7 weeks old), and 2.4 g food (8–9 weeks old). Eight hours before the training session, any remaining food was removed.

### Methodological Approach

In a first training phase (one session), mice were placed in the chambers for 15 min with the house-light off. During this time, the pellets dispenser containing 15 food pellets was open in order to familiarize mice to eat the reinforcer in the magazine. In a second phase, the lighthouse was turned on, and mice were submitted to 2 training sessions (20 min per session) in which 20 food pellets were delivered in the magazine according to a variable time schedule (mean = 60 s). On the first session, the panel was blocked in order to maintain the food dispenser open. For all other following sessions, mice had to push away the panel in front of the food dispenser to receive the food pellet. During these two phases, each hole was covered by a metal cover. In a third phase, the house light was off, the central hole (hole 5) was illuminated, and accessible for the entire duration of the session (30 min). Each time the mouse introduced its nose into the illuminated hole (nose-poke), a food pellet was provided in the magazine. This training was maintained until the mice reached at least 50 nose-pokes during the session. Subsequently, mice were trained to react to a brief visual stimulus delivered randomly in one of the five spatial locations (holes 1, 3, 5, 7, 9), as previously described (Harati et al., 2011). This starts the inter-trial interval (ITI; 5 sec standard conditions). The food reinforcer is delivered when the subject nose pokes correctly within 5 s of extinguishing the light stimulus. The following trial is initiated upon exiting the food magazine. Once the mouse correctly responded to the illuminated hole, a reward pellet was delivered. Responses to non-illuminated holes had no consequence. If the subject’s nose stings incorrectly or fails to respond within the limited 5-second timeout (considered as an omission), then the house light is turned on. If the animal pokes during the ITI, this is considered as a premature response, and the house light is illuminated. Subsequently, the subject must press the panel to start a new trial. When the basic performances are stabilized, we manipulate different tasks in order to modify the behavior of the mice. The light stimuli were presented in a pseudo-random manner (up to a maximum of 100 presentations). Initially, the stimulus duration was set to a long duration to facilitate learning (e.g., 32 s). During the subsequent sessions, the stimulus duration was progressively reduced (32, 16, 8, 4, 2, 1.8, 1.6, 1.4, 1.2, 1.0 s) until reaching the baseline value (0.8 s). The mice move to the following training level when they meet performance criteria (i.e., >70 trials, response latencies equivalent to or shorter than the stimulus duration, and >80% accuracy and <20% omissions; see Figure 4) during two consecutive sessions. The ITI duration and the length of the limited hold (the period after the extinction of the light stimulus, during which the subject can nose poke for a reward) were maintained unchanged during training (all 5 s).

Each session must be preceded by three consecutive days of stable basic performance respecting the criteria as indicated above. The altered duration of the ITI increases attentional load by disrupting the temporal predictability of the stimulus onset. Short ITI is in the range of 2–5 s, whereas long ITI is between 5 and 8 s. With an increase of the ITI duration, mice show increase in levels of premature responses that are independent of discriminative accuracy. In addition, increasing (from 0.8 to 2 s); or decreasing (from 0.8 to 0.2 s), the stimulus duration modulates attentional load.

The essential measures of performance are:

• The number of sessions at each training level defined by the stimulus duration levels.
• The accuracy of responding, defined as the number of correct commissions (correct responses/correct and incorrect responses).
• The total number of sessions to reach the baseline at 0.8 s stimulus duration.
• The number of premature nose pokes (the number of responses made during the ITI).
• The percentage of correct, incorrect, and omitted trials.
• The correct and incorrect reaction times (defined as the latency to respond in a hole after the stimulus light had been illuminated).

### Statistical Analysis

Statistical analyses were conducted with SigmaPlot 11.0 software (SigmaStat, Systat Software Inc, San Jose, CA, United States). Homoscedasticity of all data sets were confirmed by using the Levene test, and thus parametric statistics were used in all cases. For the cliff avoidance test, the chi-square and Student’s *t*-tests were used to compare between Sham and 6-OHDA groups. In addition, the three-way repeated measures ANOVA were used followed by a Tukey *post hoc* test to evaluate the difference between groups in the latent inhibition test. For each parameter of 5-CSRTT, the two-way repeated measures ANOVA followed by a Tukey *post hoc* test for multiple comparisons was performed. Results were presented as mean ± standard error of the mean (SEM), and significance was reported at *p* < 0.05.

## Results

To confirm that 6-OHDA mice display neurochemical features of ADHD and especially dopamine depletion, we examined TH-immunoreactivity (IR) in the striatum of sham and 6-OHDA adolescent mice.

We found a strong loss of TH-IR fibers in the striatum of 6-OHDA mice (Supplementary Figure S1A). Statistical analysis revealed that the 6-OHDA groups showed a significant decrease in the intensity of TH immunolabelling in comparison to sham (*p* < 0.001; Supplementary Figure S1B). In addition, TH-immunopositive area was significantly reduced in the striatum of 6-OHDA groups compared to sham (*p* < 0.001; Supplementary Figure S1C).

### Abnormal Latent Inhibition in 6-OHDA Mice

**FIGURE 2:**
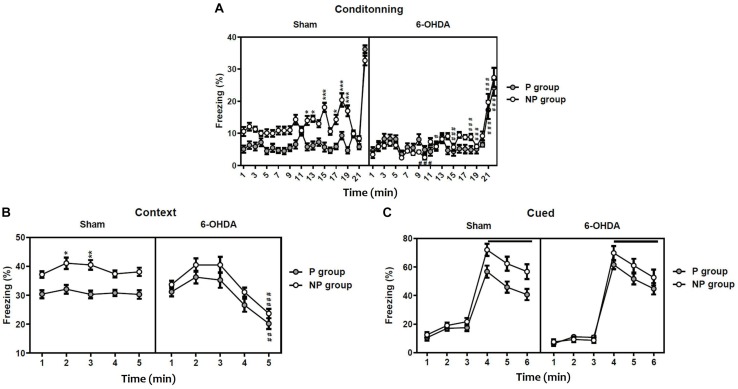
Latent inhibition in sham and 6-OHDA mice. Percentage of freezing during conditioning **(A)**, contextual testing **(B)**, and cued testing **(C)**. Data is expressed as mean ± SEM, *n* = 10 mice per group. *p < 0.05, **p < 0.01, ***p < 0.001 compared with non-preexposed group and *^#^p* < 0.05, *^##^p* < 0.01 and *^###^p* < 0.001 compared to 6-OHDA group (Three-way ANOVA followed by Tukey *post hoc* test). NP, non-pre-exposed; P, pre-exposed.

The statistical analysis with the three-way ANOVA repeated measure demonstrated a significant effect of lesion [*F*_(__1_,_396__)_ = 118.20; *F*_(__1_,_45__)_ = 13.21; *p* < 0.001 and *F*_(__1_,_54__)_ = 4.36; *p* < 0.05; respectively], exposition to the tone [*F*_(__1_,_396__)_ = 136.20; *F*_(__1_,_45__)_ = 49.57 and *F*_(__1_,_54__)_ = 33.05; *p* < 0.001; respectively] and the time [*F*_(__21_,_396__)_ = 60.96; *F*_(__4_,_45__)_ = 16.50 and *F*_(__5_,_54__)_ = 169.7; *p* < 0.001; respectively] in the freezing percentage during conditioning (Figure 2A), the contextual recall (Figure 2B) and the cued tests (Figure 2C). In addition, the interaction Sham-lesion × P-NP × time had a significant effect in conditioning [*F*_(__21_,_396__)_ = 3.51], while in the contextual recall and the cued tests [*F*_(__4_,_45__)_ = 0.14; *F*_(__5_,_54__)_ = 0.16; *p* > 0.05; respectively] had no effect.

On one hand, the *post hoc* analysis indicated that sham mice pre-exposed (P) to the tone freeze less than non-pre-exposed (NP) animals during conditioning (from session 12: *q* = 6.39, *p* < 0.05 to session 19: *q* = 9.59, *p* < 0.001; Figure 2A) and the contextual recall test (session 2: *q* = 5.20, *p* < 0.05 and session 3: *q* = 5.89, *p* < 0.01; Figure 2B), indicating significant latent inhibition in sham mice. In contrast, the freezing behavior of pre-exposed 6-OHDA mice was not different from non-exposed 6-OHDA mice during conditioning, the context test, and the cued test (*p* > 0.05; Figure 2C), suggesting a deficit of 6-OHDA mice in latent inhibition and therefore poorly sustained attention. On the other hand, the *post hoc* analysis showed a significant decrease of the freezing behavior in the 6-OHDA NP group in comparison to the sham NP group during conditioning (from session 10: *q* = 9.16, *p* < 0.001 to session 21: *q* = 8.77; *p* < 0.001; Figure 2A) and the contextual recall test at session 5 (*q* = 8.66, *p* < 0.001; Figure 2B). However, there was no significant difference between sham and 6-OHDA groups during the cued test (*p* > 0.05; Figure 2C).

### Impaired Cliff Avoidance Reaction in 6-OHDA Mice

**FIGURE 3:**
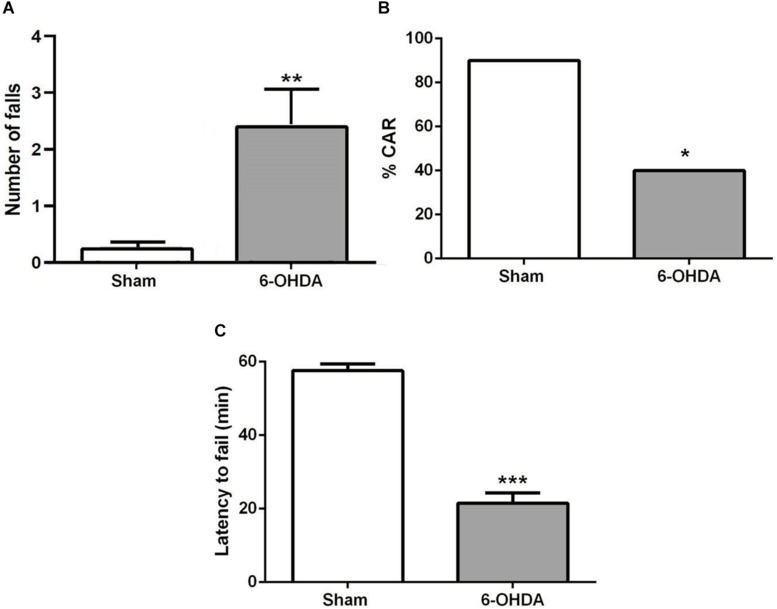
**(A)** CAR in sham and 6-OHDA mice. Values represent the percentage of CAR. *p < 0.05, compared with sham mice, *n* = 10 mice per group. **(B)** Latency from an initial placement on the platform until falling. **(C)** The latency to fail represented as mean ± SEM. ***p < 0.001 compared with sham mice, *n* = 10 mice per group. **p < 0.01 compared with sham mice.

A few minutes after the start of the test, sham mice bring their snouts close to the edge of the platform to examine it, avoiding falling. However, 6-OHDA mice repetitively investigate the edge of the platform and stay there longer. They tried to hang on the underside of the platform with their forelegs and often fell (Figure 3A; *t* = 3.0, *p* < 0.01). About 60% of 6-OHDA mice had impaired CAR during the 60 min test, while only 10% sham mice showed impaired CAR (Figure 3B; z = 2.34; *p* < *0.05*, chi-square test). In addition, 6-OHDA mice fell from the platform within 20 min test period, but none of the sham mice fell (Figure 3C; *t* = 10.74; *p* < *0.001*, Student’s *t*-test).

### Impaired Attention and Impulsivity in 6-OHDA Mice

#### The 5-CSRTT Training Effect on Body Weight

**FIGURE 4:**
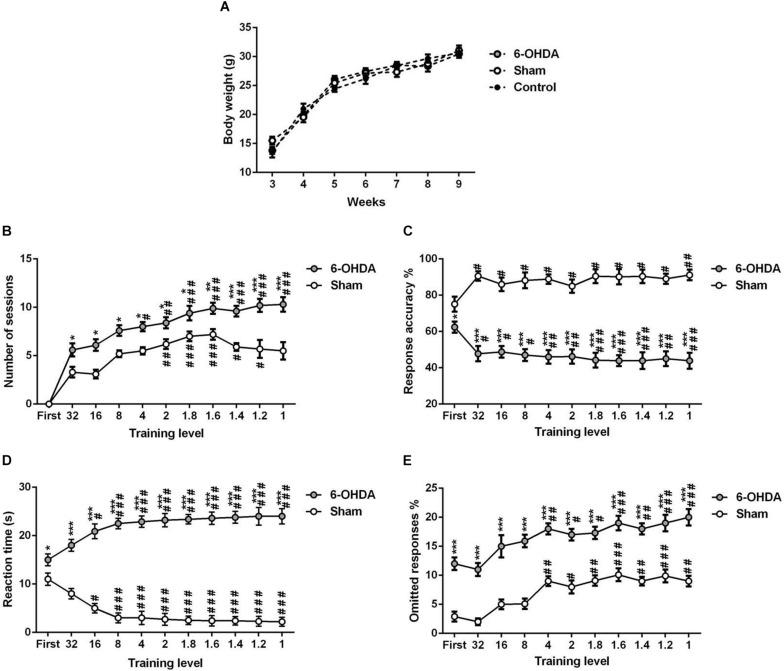
5-choice acquisition during adolescence period (ITI; 5 s standard conditions). Graphs show **(A)** body-weight of free-fed mice and food-restricted mice that underwent 5-CSRTT training, **(B)** number of sessions, **(C)** response accuracy, **(D)** correct reaction time, and **(E)** percentage of omitted responses at each training level during 5-choice acquisition in sham and 6-OHDA mice. Data is expressed as mean ± SEM, *n* = 10 mice per group. *p < 0.05, **p < 0.01, ***p < 0.001 vs. sham. *^#^p* < 0.05, *^##^p* < 0.01 and *^###^p* < 0.001 vs. 32 (Two-way ANOVA followed by Tukey *post hoc* test). ITI, intertrial interval.

Our data indicated that there is no significant change in body weight between the experimental (sham and 6-OHDA) and control (free-fed) groups of the same age [Figure 4A; *F*_(__2_,_18__)_ = 0.017, *p* > 0.05].

**FIGURE 5:**
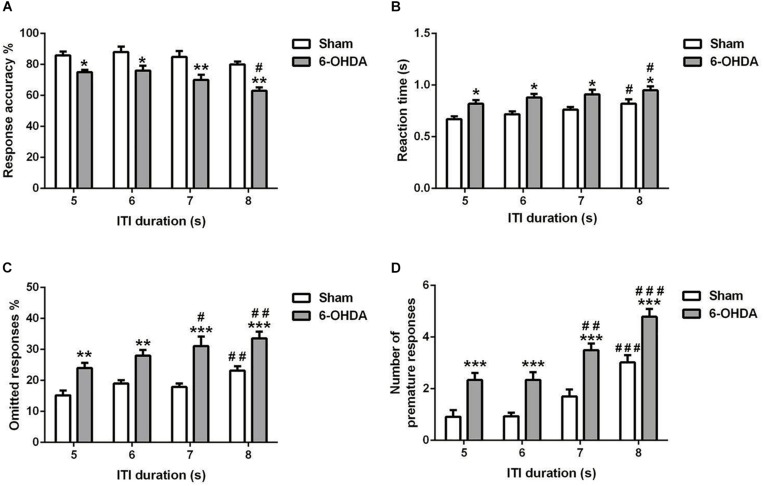
Effects of increasing the ITI on the 5-choice performance during young adult period. Graphs show **(A)** response accuracy, **(B)** correct reaction time, **(C)** omission errors, and **(D)** premature responses at each of the ITI durations in sham and 6-OHDA mice. Data is expressed as mean ± SEM, *n* = 10 mice per group. *p < 0.05, **p < 0.01, ***p < 0.001 vs. sham; ^#^*p* < 0.05, ^##^*p* < 0.01 and ^###^*p* < 0.001 vs. ITI 5 s (Two-way ANOVA followed by Tukey *post hoc* test.

**FIGURE 6:**
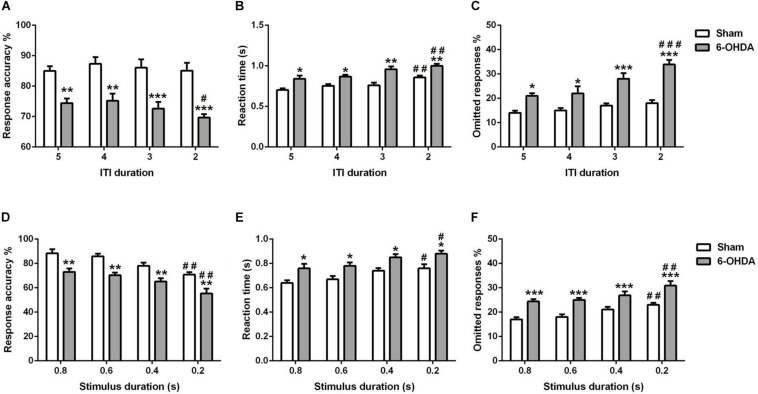
Effects of decreasing the ITI **(A–C)** and SD **(D–F)** on 5-choice performance during young adult period. Graphs show **(A,C)** response accuracy, **(B,D)** correct reaction time, and **(C,F)** omission errors. Data is expressed as mean ± SEM, *n* = 10 mice per group. *p < 0.05, **p < 0.01, ***p < 0.001 vs. sham; ^#^*p* < 0.05, ^##^*p* < 0.01, and ^###^*p* < 0.001 vs. ITI 5 s or SD 0.8 s (Two-way ANOVA followed by Tukey *post hoc* test).

Listed below are the results obtained for each of the commonly used tasks manipulations (Figures 4–6).

#### 5-Choice Acquisition

We tested the different parameters of the 5-CSRTT acquisition in adolescent mouse. The stimulus duration (i.e., training level) is progressively shortened while the following parameters are assessed: number of sessions to maintain stable performance (Figure 4B), response accuracy (Figure 4C), the time needed for a correct reaction (Figure 4D), and percentage of omissions (Figure 4E). Two-way ANOVA repeated measures analysis was performed with lesion and training level as main factors. During the training, the results showed that the number of sessions, response accuracy, reaction time and omitted responses were affected by the lesion [*F*_(__1_,_9__)_ = 192.70; *F*_(__1_,_9__)_ = 404.30; *F*_(__1_,_9__)_ = 450.10 and *F*_(__1_,_9__)_ = 354.20; *p* < 0.001; respectively] and the training level [*F*_(__9_,_81__)_ = 11.18; *F*_(__10_,_90__)_ = 6.01; *F*_(__10_,_90__)_ = 12.98 and *F*_(__10_,_90__)_ = 14.34, *p* < 0.001; respectively]; while the interaction between the two factors was not affected [*F*_(__9_,_81__)_ = 1.67; *F*_(__10_,_90__)_ = 0.09; *F*_(__10_,_90__)_ = 0.02 and *F*_(__10_,_90__)_ = 0.34; *p* > 0.05; respectively]. In fact, the *post hoc* analysis showed a significant increase of the number of sessions in the 6-OHDA group in comparison to the sham group in all training levels (Table 1). Moreover, the reaction time and omitted responses were significantly increased in 6-OHDA mice as compared to the sham mice (Table 1). Meanwhile, the accuracy of responses was reduced significantly in the 6-OHDA group compared to the sham group (Table 1).

**TABLE 1:** Statistical analysis of the mice groups effects (Sham/6-OHDA) and training level on number of sessions, response accuracy, reaction time, and omitted responses in 5-choice acquisition.

Parameters of the 5-CSRTT acquisition	Stimulus duration (seconds)	Groups		Two-way ANOVA with repeated measures	Tukey *post hoc* 6-OHDA vs. Sham
				
		Sham	6-OHDA		
**Number of**	First (60)	—	—		—
** sessions**	32	3.30 ± 0.53 s	5.60 ± 0.67 s	Group effect:	*q* = 3.12*
	16	3.10 ± 0.45 s	6.10 ± 0.60 s	*F*_(1,9)_ = 192.7***	*q* = 4.08*
	8	5.20 ± 0.35 s	7.60 ± 0.56 s	Level effect:	*q* = 3.26*
	4	5.50 ± 0.37 s	8.00 ± 0.47 s	*F*_(9,81)_ = 11.18***	*q* = 3.40*
	2	6.20 ± 0.51 s	8.40 ± 0.58 s	Interaction:	*q* = 2.99*
	1.8	7.00 ± 0.49 s	9.40 ± 0.74 s	*F*_(9,81)_ = 1.67	*q* = 3.26*
	1.6	7.20 ± 0.55 s	9.90 ± 0.58 s		*q* = 3.67**
	1.4	5.90 ± 0.40 s	9.60 ± 0.54 s		*q* = 5.03***
	1.2	5.70 ± 0.91 s	10.20 ± 0.67 s		*q* = 6.12***
	1	5.50 ± 0.90 s	10.30 ± 0.74 s		*q* = 6.52***
**Response**	First (60)	75.10 ± 4.16 s	62.40 ± 3.00 s		*q* = 3.17*
**accuracy**	32	90.60 ± 2.62 s	47.80 ± 4.25 s	Group effect:	*q* = 10.70***
	16	86.00 ± 3.68 s	48.80 ± 3.20 s	F_(1,9)_ = 404.3***	*q* = 9.30***
	8	88.20 ± 4.32 s	47.00 ± 3.33 s	Level effect:	*q* = 10.30***
	4	88.90 ± 2.61 s	46.00 ± 3.75 s	F_(10,90)_ = 0.098	*q* = 10.73***
	2	85.00 ± 3.63 s	46.20 ± 3.90 s	Interaction:	*q* = 9.70***
	1.8	90.50 ± 3.82 s	44.20 ± 4.09 s	F_(10,90)_ = 6.00***	*q* = 11.58***
	1.6	90.20 ± 4.23 s	43.90 ± 2.99 s		*q* = 11.58***
	1.4	90.40 ± 3.69 s	43.90 ± 4.60 s		*q* = 11.63***
	1.2	89.10 ± 2.77 s	45.00 ± 4.10 s		*q* = 11.03***
	1	91.20 ± 2.87 s	43.90 ± 4.41 s		*q* = 11.83***
**Reaction time**	First (60)	11.00 ± 1.25 s	15.00 ± 1.22 s		*q* = 2.49*
	32	8.00 ± 1.08 s	18.00 ± 1.24 s	Group effect:	*q* = 6.22***
	16	5.00 ± 0.97 s	20.90 ± 1.50 s	F_(1,9)_ = 450.1***	*q* = 9.90***
	8	3.00 ± 1.03 s	22.50 ± 1.07 s	Level effect:	*q* = 12.14***
	4	3.00 ± 1.38 s	22.90 ± 1.18 s	F_(10,90)_ = 0.016	*q* = 12.39***
	2	2.70 ± 1.21 s	23.20 ± 1.37 s	Interaction:	*q* = 12.76***
	1.8	2.50 ± 0.88 s	23.40 ± 0.95 s	F_(10,90)_ = 12.98***	*q* = 13.01***
	1.6	2.40 ± 1.08 s	23.60 ± 1.29 s		*q* = 13.20***
	1.4	2.40 ± 0.87 s	23.80 ± 1.20 s		*q* = 13.32***
	1.2	2.30 ± 1.00 s	24.00 ± 1.81 s		*q* = 13.51***
	1	2.20 ± 0.92 s	24.00 ± 1.59 s		*q* = 13.57***
**Omitted**	First (60)	2.90 ± 0.86 s	12.00 ± 1.08 s		*q* = 6.23***
**responses**	32	2.00 ± 0.59 s	11.00 ± 1.10 s	Group effect:	*q* = 6.16***
	16	5.00 ± 0.81 s	15.00 ± 1.93 s	*F*_(1,9)_ = 354.2***	*q* = 6.84***
	8	5.10 ± 0.91 s	15.90 ± 1.07 s	Level effect:	*q* = 7.39***
	4	9.00 ± 0.83 s	18.00 ± 0.96 s	*F*_(10,90)_ = 14.34***	*q* = 6.16***
	2	8.00 ± 1.11 s	17.00 ± 0.98 s	Interaction:	*q* = 6.16***
	1.8	9.10 ± 0.91 s	17.30 ± 1.05 s	*F*_(10,90)_ = 0.34	*q* = 5.61***
	1.6	10.10 ± 1.11 s	19.00 ± 1.23 s		*q* = 6.09***
	1.4	9.00 ± 0.76 s	18.00 ± 0.96 s		q = 6.16***
	1.2	9.90 ± 1.10 s	19.00 ± 1.40 s		*q* = 6.16***
	1	9.00 ± 0.90 s	20.00 ± 1.41 s		*q* = 7.53***

The values reported are the mean ± SD (*n* = 10 per group). *p < 0.05 and ***p < 0.001 refers to the sham versus 6-OHDA group comparison.

#### Long ITI

5-choice performance was assessed upon increasing ITI in young adult mouse. In all the parameters investigated [e.g., response accuracy (Figure 5A), reaction time (Figure 5B), omitted (Figure 5C), and premature responses (Figure 5D)], the two-way ANOVA repeated measures (lesion and ITI duration) revealed a significant effect of lesion [*F*_(__1_,_9__)_ = 62.80; *F*_(__1_,_9__)_ = 77.96; *F*_(__1_,_9__)_ = 36.24 and *F*_(__1_,_9__)_ = 105.9; *p* < 0.001; respectively] and ITI duration [*F*_(__3_,_27__)_ = 7.43; *F*_(__3_,_27__)_ = 4.23, *p* < 0.05; *F*_(__3_,_27__)_ = 9.20 and *F*_(__3_,_27__)_ = 33.14, *p* < 0.001; respectively] at the longest ITI duration, with no effect of the interaction of lesion × ITI duration [*F*_(__3_,_27__)_ = 0.46; *F*_(__3_,_27__)_ = 0.08; *F*_(__3_,_27__)_ = 0.78 and *F*_(__3_,_27__)_ = 0.47, *p* > 0.05; respectively). When the ITI was lengthened from 5 to 8 s, a significant increase in reaction time (*p* < 0.05), omitted (*p* < 0.01 and *p* < 0.001), and premature responses (*p* < 0.001) was observed in 6-OHDA mice (Figures 5B–D). Meanwhile, the 6-OHDA animals were significantly less accurate than sham animals from 5 (*p* < 0.05) to 8 s (*p* < 0.01) (Figure 5A). In addition, we did not observe difference on accuracy in sham mice when the ITI increased (*p* > 0.05); while at 8 s, the response accuracy was decreased significantly (*p* < 0.05) in 6-OHDA mice as compared to that observed at 5 s. The reaction time of both sham and 6-OHDA mice were significantly increased (*p* < 0.05) at 8 s as compared to 5 s. Indeed, the omitted and premature responses of the 6-OHDA group were increased significantly at 7 s (*p* < 0.05 and *p* < 0.01; respectively) and 8 s (*p* < 0.01 and *p* < 0.001; respectively) as compared to 5 s. However, those parameters were increased significantly only at 8 s (*p* < 0.01 and *p* < 0.001; respectively) as compared to 5 s. in sham mice.

#### Reduced ITI

We measured the influence of decreasing the ITI in young adult mouse on the 5-choice performance [e.g., response accuracy (Figure 6A), response time (Figure 6B), and percentage of omission (Figure 6C)]. Two-way ANOVA repeated measures revealed that accuracy responses, reaction time and omitted responses were different between groups [*F*_(__1_,_9__)_ = 285.80, *F*_(__1_,_9__)_ = 69.00 and *F*_(__1_,_9__)_ = 146.9, *p* < 0.001; respectively] and varied with ITI duration [*F*_(__3_,_27__)_ = 1.80, *p* < *0.05*; *F*_(__3_,_27__)_ = 12.42 and *F*_(__3_,_27__)_ = 9.47, *p* < 0.001; respectively]; while the interaction of group and ITI duration had no effect [*F*_(__3_,_27__)_ = 0.51; *F*_(__3_,_27__)_ = 0.73 and *F*_(__3_,_27__)_ = 2.72, *p* > 0.05; respectively]. The *post hoc* analysis showed a reduction of response accuracy in 6-OHDA group (5–4 s: *p* < 0.01 and 3–2 s: *p* < 0.001) at all ITI duration (5–2 s) as compared to the sham group. Meanwhile, from 5 until 2 s of ITI duration, the reaction time (5–4 s: *p* < 0.05 and 3–2 s: *p* < 0.01) and omitted responses (5–4 s: *p* < 0.05 and 3–2 s: *p* < 0.001) of the 6-OHDA group were higher when compared to the sham group (Figure 6). At 2 s ITI duration, the 6-OHDA mice were less accurate (*p* < 0.05), reacted more slowly (*p* < 0.01) and made more omission errors (*p* < 0.001) in comparison to the 5 s ITI duration. By contrast, only the reaction time at 2 s was increased (*p* < 0.01) in sham mice, while the accuracy and omitted responses at any ITI duration were not different from those of 5 s (Figure 6).

#### Reduced Stimulus Duration

The effect of shortening the stimulus duration was evaluated on the same parameters as above [e.g. response accuracy (Figure 6D), reaction time (Figure 6E), and omitted response (Figure 6F)]. For all the parameters used, there is an effect of lesion [*F*_(__1_,_9__)_ = 75.36; *F*_(__1_,_9__)_ = 67.06; *F*_(__1_,_9__)_ = 63.40, *p* < 0.001; respectively] and stimulus duration [*F*_(__3_,_27__)_ = 14.21; *F*_(__3_,_27__)_ = 9.28; *F*_(__3_,_27__)_ = 9.33, *p* < 0.001; respectively]; with no effect of the interaction between those two factors [*F*_(__3_,_27__)_ = 0.09; *F*_(__3_,_27__)_ = 0.02; *F*_(__3_,_27__)_ = 0.31, *p* > 0.05; respectively]. The *post hoc* analysis showed that response accuracy was decreased significantly in both groups at 0.2 s of SD as compared to 0.8 s (*p* < 0.01; Figure 6D). Furthermore, the reaction time was increased at shortest SD (0.2 s) as compared to longest SD (0.8 s) in sham and 6-OHDA mice (*p* < 0.05; Figure 6E). However, the omitted response was increased when the 0.2 s of SD compared to 0.8 s in both of the groups (*p* < 0.01; Figure 6F).

## Discussion

In the present study, we demonstrated for the first time a disturbance in the latent inhibition and a poorly sustained attention in adolescent-like 6-OHDA mice, suggesting that not only adult, but also juvenile 6-OHDA mice show behavioral alterations associated with ADHD. Indeed, an alteration in the latent inhibition reflects a deficiency in selective attention, and a pathologically high tendency to replace or exchange the non-contingent association previously learned with the appropriate CS - US response (Hemsley, 1993). Even if no theory takes this phenomenon into account (Escobar et al., 2002), a disturbance in the latent inhibition is closely linked with the inattention/impulsivity distinctive of ADHD. In our study, 6-OHDA juvenile mice easily managed to associate the CS and US, as showed by the non-preexposed groups. This assumes that the impairment of the latent inhibition is probably not due to alteration in associative learning, but rather indicates deficit in selective attention.

In addition, we showed that 6-OHDA juvenile mice present highly impulsive behavior in the CAR test. However, optimizing the actions of animals requires serious control of their impulses. This control seems to be linked to distinct neuronal and neurochemical systems (Fineberg et al., 2010; Whelan et al., 2012). In addition, the CAR impairments seen in juvenile 6-OHDA mice may be contributed to repetitive exploratory behaviors due to persevering motor behavior.

In ADHD patients, both the attentional and impulse control deficits can be proved by the continuous performance task (CPT). In fact, because of their attention deficit, subjects with ADHD have slower and more variable reaction times and make more errors of omission (Epstein et al., 2003; Winstanley et al., 2006). These patients also exhibit reduced behavioral inhibition demonstrated by their high score of error commissions. In addition, high levels of impulsivity are determined in ADHD patients by numerous tests (Solanto, 1998; Winstanley et al., 2006). 5-CSRTT is a test usually used in rodents that can show behavioral inhibition aspect (Carli et al., 1983). In fact, the 5-CSRTT (Robbins, 2002) and 3-choice serial reaction time task (3-CSRTT) (Tsutsui-Kimura et al., 2009) has been usually used to study impulsivity in adult rats and mice. In addition, the main disadvantage of 5-CSRTT is that it lasts months to finished the mice training and reach stable performance levels. Another disadvantage of the 5-CSRTT is the mild food restriction used to motivate task performance (Asinof and Paine, 2014). In fact, this motivation to respond decreases as the session continues, since the subjects become full, and consequently their performance can be affected (Grottick and Higgins, 2002). However, pre-feeding subjects before the test is a way to determine whether satiety is involved in the effects of a particular manipulation (Grottick and Higgins, 2002; Bari et al., 2008; Nemeth et al., 2010). By cons, several methods have been recently developed to assess attention and impulsivity in mice (Remmelink et al., 2017; Bruinsma et al., 2019). The author’s performed their method with particular equipment (The CombiCage and self-paced 5-CSRTT protocol) that allowed mice to learn fast. In their protocols, mice had 24-h/day continuous task access, during which they could earn unlimited food rewards based on tasks, and achieve task progress at their own pace. This free access to the task can induce an automation of the performance or usual responses; which could make the performance on the standard task unchangeable to particular manipulations.

In this study, we adapted the 5-CSRTT protocol in order to assess attention and inhibitory control during the adolescence-like period in mice. First, we controlled the food restriction in the youngest mice, to allow them to have a normal growth and to be sufficiently motivated to accomplish tasks. Thus, we have shown that this diet adopted during all the training phases allowed an almost normal development (Figure 4). In addition, the duration of training was similar to that of adult mice reported in our previous study (Bouchatta et al., 2018). These results prove that the diet applied to young mice induced sufficient motivation to acquire the tasks, without stunting. Second, we reduced the number of sessions needed to terminate the training. Third, we carried out training sessions without sanction, allowing many opportunities to discover a brief light stimulus during the accustomed training procedure. In our adapted 5-CSRTT protocol, the mice were conditioned to successfully respond to a 1 s stimulus after just 6 weeks (Figure 4), which is shorter than other procedures (Humby et al., 2005). Our data confirmed that sham and 6-OHDA mice learned the complex 5-CSRTT task. Both groups made more than 50% of correct responses at the first stage of training (Figure 4). These results agree with a similar protocol using a 3-choice serial reaction time task (Sasamori et al., 2018).

Attention is most often evaluated using the percentage of response omissions (Robbins, 2002; Amitai and Markou, 2011) as well as by the responses accuracy (Robbins, 2002; Bari et al., 2008; Amitai and Markou, 2011). In addition, it has been shown that this accuracy is not affected by locomotor ability, motivation or sedation (Asinof and Paine, 2014). For the first time, we demonstrated that sham and 6-OHDA adolescent-like mice exhibit different performance in the 5-CSRTT, especially when attentional demands are high.

This difference between sham and 6-OHDA lesioned mice observed throughout the session, would underlie the presence of a deficit of selective attention and difficulties in keeping sustained attention, similar to human situations. Various modifications of the parameters raise the attention demands of the 5-CSRTT, e.g. shortening the ITI or decreasing the duration of the stimulus (Figure 6), and indicate that young adult 6-OHDA mice exhibited a larger drop in precision when attention was tested. Response inhibition can be easily tested in the 5-CSRTT to assess impulsivity by increasing the extent of the ITI. Premature responses are those occurring during the ITI before the stimulus presentation, and lengthening the ITI leads to a consistent increase in the number of these premature responses. Premature responses are a form of impulsive behavior and represent failures in impulse control (Robbins, 2002; Bari et al., 2008) that reflects a lack of response inhibition (Evenden, 1999; Robbins, 2002). Taken together, we demonstrated that young adult 6-OHDA mice showed a disturbance in the inhibitory control on the 5-CSRTT, as expressed by the increase of premature responses during the inter-trial interval task (Figure 5).

ADHD result from dopamine (DA) system dysfunction of certain cortical structures such as the prefrontal cortex, mainly the right-medial side (Sullivan and Brake, 2003), and subcortical areas, particularly the nucleus accumbens and the striatum (Russell et al., 2005). Neonatal 6-OHDA animal models showed a clear functional impairment of the dopaminergic system (Shaywitz et al., 1976; Luthman et al., 1989; Zhang et al., 2001; Moran-Gates et al., 2005). In addition, in rodents, the postnatal development of the nigrostriatal neuronal DAergic activity described during the first 2 weeks is more important for the final development of excitatory synapses in the corticostriatal pathway by reducing the glutamate release (Choi and Lovinger, 1997). Consequently, an increase in glutamatergic transmission would be obtained during a selective disturbance of this DAergic pathway during this critical period (Tang et al., 2001). It is therefore conceivable that the behavioral characteristics of ADHD could result from an alteration in dopaminergic modulation of neurotransmission in the cortico-striato-thalamo-cortical circuits.

## Conclusion

The present study demonstrated defects in latent inhibition and poorly sustained attention, suggesting that 6-OHDA mice display supplementary behavioral impairments associate with ADHD. Moreover, 6-OHDA mice show inadequately impulsive behavior in the CAR test. We can then suppose that attentional deficits highlighted by 5-CSRTT could in part be due to this impulsive behavioral disturbance. However, we were able successfully to surmount the limitations of in effect, our modified 5-CSRTT protocol prevented growth disruptions and significantly reduced the training duration, allowing us to assess attention and impulsivity in mice adolescence. Therefore, it is now possible to assess parameters of neurodevelopmental disorders in rodent models in conditions that are close to the human situation. The 6-OHDA mouse model will be useful in understanding and supporting the basic neurobiological mechanisms of this heterogeneous, and complex disorder at different periods of neurodevelopment.

## Data Availability Statement

The datasets generated for this study are available on request to the corresponding author.

## Ethics Statement

The animal study was reviewed and approved by the Council Committee of Research Laboratories of the Faculty of Sciences, Cadi Ayyad University. All procedures were conducted in accordance with the approved institutional protocols and within the provisions for animal care and use prescribed in the scientific procedures on living animals, European Council Directive (EU2010/63).

## Author Contributions

OB, SB-M, ML, and MB conceived the experiments. OB, HM, and SB-M performed the experiments. OB, HM, SB-M, ML, and MB analyzed the data. OB, SB-M, ML, and MB wrote the manuscript.

## Conflict of Interest

The authors declare that the research was conducted in the absence of any commercial or financial relationships that could be construed as a potential conflict of interest.
