# Supplementary material for: Neonatal 6-OHDA Lesion Model in Mouse Induces Cognitive Dysfunctions of Attention-Deficit/Hyperactivity Disorder (ADHD) During Young Age
Source: Front Behav Neurosci. 2020 Feb 26;14:27. doi: 10.3389/fnbeh.2020.00027 (PMC7054716; doi:10.3389/fnbeh.2020.00027)
Supplement: Supplementary file 1 [file Data_Sheet_1.docx]

**Supplementary Method:**

Five Swiss male sham and five Swiss male 6-OHDA mice were perfused through the ascending aorta with cold fixative containing 4% paraformaldehyde diluted in phosphate buffer (0.1 M). Brains cord were rapidly dissected and postfixed in the same fixative for 2 hours. Tissues were then incubated in phosphate-buffered saline (PBS, pH 7.4) containing 12% sucrose and sodium azide at 4°C overnight. The brains were frozen and cut at 18µm in a cryostat (Leica, Wetzlar, Germany). Sections were collected in cell wells and the subsequent steps were carried out on free floating sections.

Sections were rinsed in PBS, and incubated in PBS containing 1% bovine serum albumin (BSA; Sigma) (PBS-BSA) for 30 minutes at room temperature. Sections were then incubated overnight at 4°C in PBS-BSA with sheep anti-TH (1/500, RD System (Bio Techne) AF7566-SP) antibody. They were then rinsed 3 times 10 minutes in PBS containing 0.05% Tween 20 (PBT). Alexa 488-conjugated donkey anti-sheep (Thermo Fisher Scientific, n°A11015) (1:500 in PBS-BSA) was then applied to the sections for 2 hours at room temperature. Sections were rinsed and finally mounted in Fluoroshield containing DAPI for nucleus visualization (Sigma, n° F6057).

Specificity of the antibodies used was determined by the manufacturer and in our hands by omitting the primary antibody. Immunostaining was visualized under a SPE confocal microscope (Leica Microsystems). Images to be compared were collected during the same session using identical scanning settings. They were then imported into “ImageJ” free software (version 1.42q) (NIH, Bethesda, MA) for quantitative analysis. Background was subtracted by thresholding. The mean gray level corresponding to fluorescence intensity, and the immunolabeled area, were measured in sham and 6-OHDA mice (2 to 4 sections per mouse, 4 mice per condition) (Zaki et al., 2018). Results were expressed as a percentage of the intensity, or immunolabeled area, in sham animals. Statistical analysis was performed with student t-test.

**Supplementary reference:**

Zaky A, Bouali-Benazzouz R, Favereaux A, Tell G, Landry M. (2018) APE1/Ref-1 redox function contributes to inflammatory pain sensitization. Exp. Neurol. 307:1-11.

**Supplementary figure legends:**

**Figure S1:** TH immunohistochemistry in the striatum of sham and 6-OHDA mice. (A) Representative micrographs of striatum. (B) Intensity of TH immunolabelling. (C) TH immunolabelled area. Values are represented as mean ± SEM ( ****p* < *0*.*001*; t-test; n = 9/4).
