# Supplementary figures and images for: Neonatal 6-OHDA Lesion Model in Mouse Induces Cognitive Dysfunctions of Attention-Deficit/Hyperactivity Disorder (ADHD) During Young Age
Source: Front Behav Neurosci. 2020 Feb 26;14:27. doi: 10.3389/fnbeh.2020.00027 (PMC7054716; doi:10.3389/fnbeh.2020.00027)

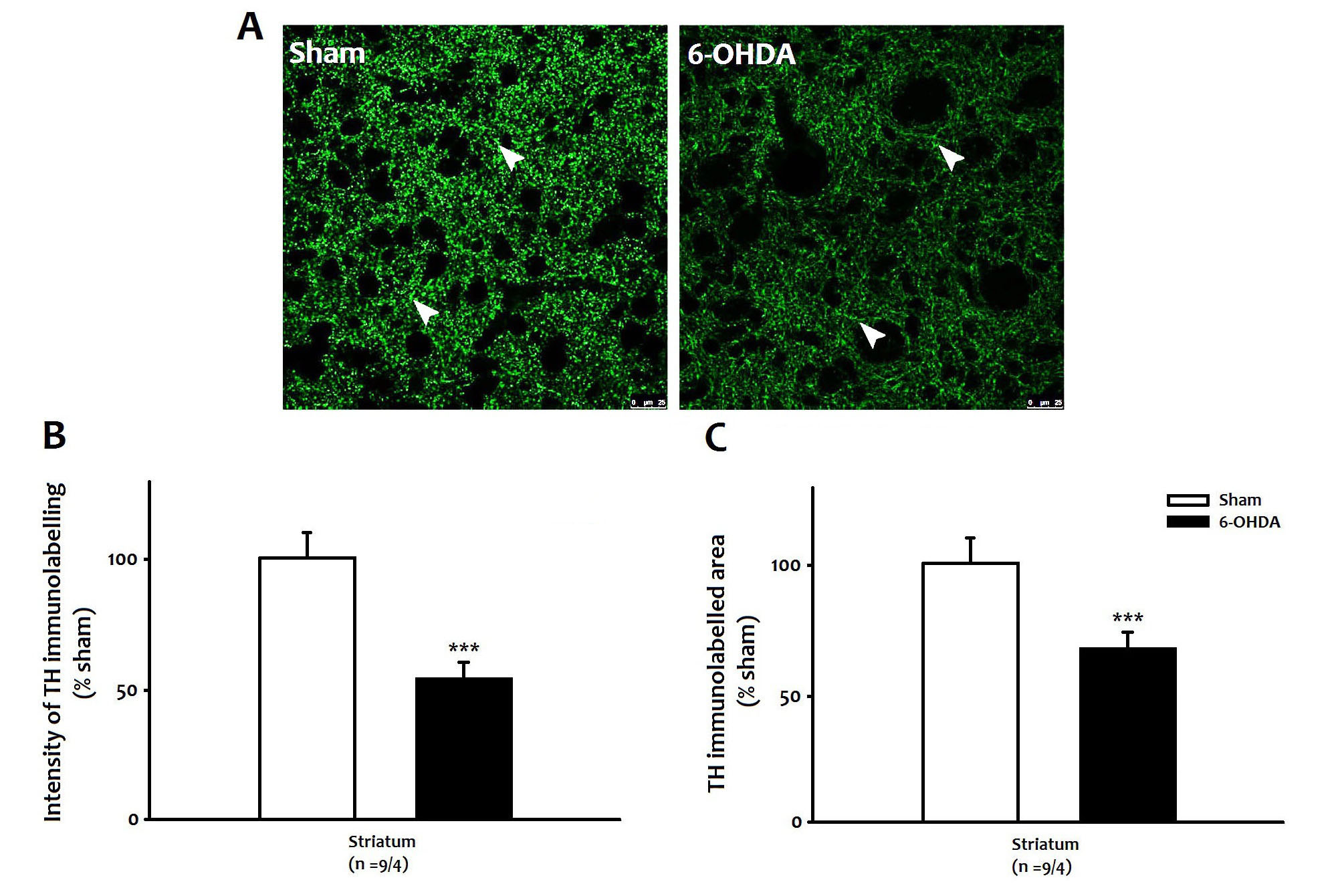

Supplement: Supplementary file 2 [file Image_1.jpeg]
